# Multifocal Emphysematous Osteomyelitis of the Spine and Pelvis

**DOI:** 10.5334/jbsr.4237

**Published:** 2026-02-26

**Authors:** Diogo Carvalho, Luís Monteiro Cabral, Raquel Dias

**Affiliations:** 1 Instituto Português de Oncologia de Lisboa Francisco Gentil, Lisboa, Portugal

**Keywords:** emphysematous osteomyelitis, spine, iliac bone, MRI, CT

## Abstract

*Teaching point:* Detection of intraosseous gas with a bubbly or irregular pattern on imaging should raise a strong suspicion for emphysematous osteomyelitis and prompt urgent antimicrobial treatment, particularly in immunocompromised patients without a history of trauma or surgery.

## Case History

A 45-year-old man with advanced anaplastic thyroid carcinoma, receiving corticosteroid therapy for dysphagia due to oesophageal involvement, presented to the emergency department with a 3-day history of fever, back pain, localized pain in the right iliac region and difficulty walking. Laboratory evaluation revealed elevated inflammatory markers, including a C-reactive protein level of 15 mg/dL (normal < 5 mg/dL). Haemocultures were positive for *Clostridium perfringens*.

**Figure 1:**
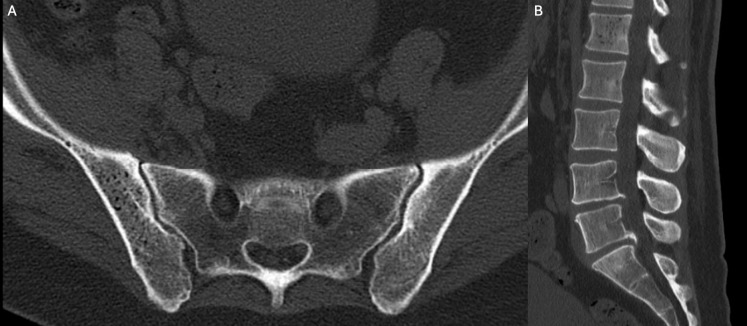
Axial CT of the pelvis **(A)** and sagittal CT reformation of the lumbar spine **(B)** demonstrate multiple low-attenuation foci in keeping with intraosseous gas within the right iliac wing and the L1 vertebral body, respectively.

**Figure 2:**
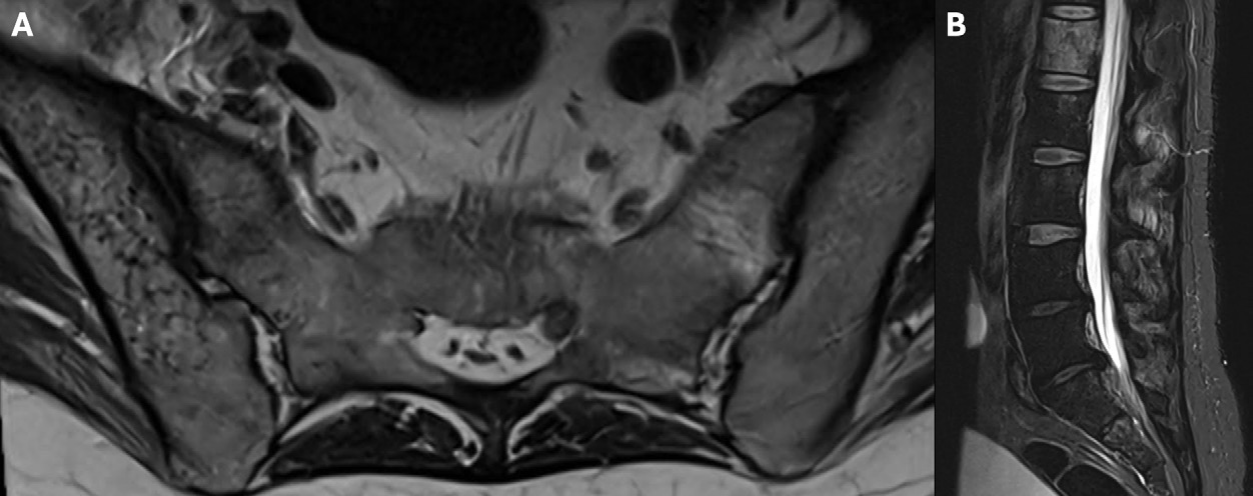
Axial T2-weighted **(A)** and sagittal STIR **(B)** MRI sequences confirm the presence of multiple hypointense foci in keeping with intraosseous gas within the right iliac wing and the L1 vertebral body. The L1 vertebral body also demonstrates diffuse increased signal compared with adjacent vertebrae, compatible with bone marrow oedema.

Spine computed tomography (CT) demonstrated irregularly distributed intraosseous gas within the right iliac wing (Figure 1A) and the L1 vertebral body (Figure 1B). Axial T2-weighted (Figure 2A) and sagittal short tau inversion recovery (STIR) (Figure 2B) magnetic resonance imaging (MRI) sequences confirmed these findings, demonstrating multiple hypointense foci consistent with intraosseous gas. The L1 vertebral body additionally showed a diffuse hyperintense signal compared with adjacent vertebrae, compatible with bone marrow oedema. No extension to the intervertebral discs or epidural space was identified.

**Figure 3:**
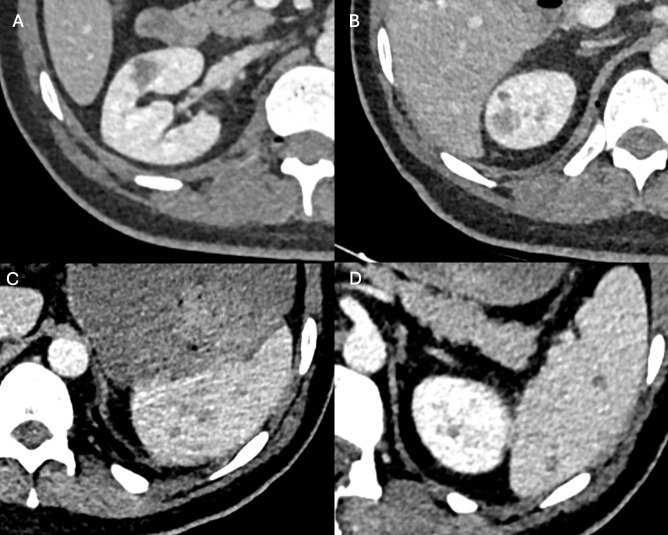
Axial contrast-enhanced CT of the abdomen demonstrates multiple small, ill-defined hypodense lesions within the right kidney **(A, B)** and spleen **(C, D)**, suspicious for septic emboli.

Based on these findings, multifocal osteomyelitis was suspected, and antibiotic therapy was initiated. To assess for additional infectious sites, contrast-enhanced CT of the thorax, abdomen and pelvis was performed, revealing multiple additional hypodense lesions in the right kidney (Figure 3A-B) and spleen (Figure 3C-D), suspicious for further involvement.

These findings were consistent with multifocal emphysematous osteomyelitis due to *Clostridium perfringens* with suspected renal and splenic septic emboli.

## Comment

Emphysematous osteomyelitis is a rare but life-threatening form of bone infection caused by gas-forming microorganisms, most commonly anaerobes and members of the Enterobacteriaceae family. The presence of intraosseous gas in the absence of trauma, penetrating wounds, orthopaedic or other surgical procedures is highly suggestive of this diagnosis and should prompt urgent evaluation. The condition predominantly occurs in immunocompromised patients, including those with diabetes mellitus, receiving corticosteroid therapy, affected by haematological disorders, malignancies, HIV infection or alcohol abuse [1].

Spinal involvement is particularly uncommon and carries a high risk of morbidity. CT is particularly useful in detecting intraosseous gas and defining its extent, typically demonstrating multiple irregular or ‘bubbly’ gas collections. MRI complements CT by better depicting associated bone marrow oedema, soft tissue extension and potential neurological complications. The differential diagnosis of intraosseous gas includes degenerative changes, osteonecrosis, fractures and neoplastic processes; however, the coexistence of systemic inflammatory signs and a characteristic gas distribution pattern strongly favours an infectious aetiology [1].

In the present case, multifocal emphysematous osteomyelitis involving the spine and pelvis was identified in an immunocompromised patient with *Clostridioides perfringens* bacteraemia, with suspected septic emboli to the spleen and kidney. Early recognition of this imaging pattern allowed prompt initiation of antimicrobial therapy, which is crucial given the high reported mortality associated with emphysematous osteomyelitis.

Despite antibiotic treatment, the patient’s condition deteriorated, leading to septic shock and death.
